# Assessing the impact of a health education outreach project on cervical cancer awareness among Vietnamese-American women in San Diego

**DOI:** 10.3934/publichealth.2022038

**Published:** 2022-06-28

**Authors:** Eduardo Fricovsky, Mudassar Iqbal Arain, Binh Tran, Phuong Thao Nguyen, Tuyet Phan, Natalie Chang

**Affiliations:** 1 SKAGGS, School of Pharmacy and Pharmaceutical Sciences, UC San Diego, USA; 2 Department of Pharmacy Practice, Faculty of Pharmacy, University of Sindh, Jamshoro, Pakistan

**Keywords:** human papillomavirus, cervical cancer, pharmacy, Vietnamese-American, San Diego

## Abstract

The objective of this study was to assess the rate of effectiveness of cervical cancer awareness outreach among Vietnamese women in San Diego, USA. In collaboration with different community partners, educational seminars were hosted by student pharmacists in the Vietnamese community. We hypothesized that the seminars would increase cervical cancer awareness and encourage a positive outlook on obtaining annual Pap smears and HPV vaccines. The study design included pre- and post-intervention assessment surveys in either Vietnamese or English language. The surveys were administered to Vietnamese women who participated in the seminars. Eight seminars were hosted at local health fairs in San Diego. A total of 120 Vietnamese women participated in the seminars. Our study showed that educational seminars significantly improved the knowledge about cervical cancer, Pap smears and HPV vaccines. By comparing the pre- and post-intervention surveys, we observed an improvement in knowledge about cervical cancer (61% vs 93%, p < 0.001) and a positive change in the attitude towards obtaining a Pap smear within a year following the educational intervention (57% vs. 78%, p < 0.002). Therefore, we concluded that the educational health outreach seminars presented by student pharmacists are an effective educational model to help improve knowledge about cervical cancer and prevention among Vietnamese women.

## 1. Introduction

Historically, cervical cancer has been the leading cause of cancer-related death for women in the United States of America. Due to advancements in the early detection and treatment options, the prevalence and rate of mortality have decreased in the USA, but they are still high in some subgroups, particularly among Vietnamese women [1],[2]. According to the latest data from the Center for disease control and prevention, more than 14,000 new cases were reported in 2020 and more than 4000 women died that year from cervical cancer [3]. The World Health Organization reported that cervical cancer is the 4^th^ leading cause of death among women worldwide [4].

Cervical cancer is preventable if detected at an early stage. Pap smear screening and HPV vaccines are effective preventative measures [5],[6]. According to the American Cancer Society guidelines, women aged 21–65 years should obtain a Pap smear every 3 years [7]. A low level of knowledge about HPV and cervical cancer was reported among women in qualitative studies conducted in four developing countries (i.e., India, Peru, Uganda and Vietnam) [8]. Despite the proven benefit of Pap tests, Vietnamese-American Women (VAM) are among the ethnic groups with the lowest rates of Pap tests and subsequently high rates of invasive cervical cancer according to the Surveillance Epidemiology and End Results Program in the USA [9],[10].

According to the reviewed literature, 37–74% of Vietnamese women have had a pap smear [9]. Studies have been conducted showing the effectiveness of educational seminars hosted by Vietnamese lay health workers and media interventions (e.g., newspapers, educational articles and radio talk shows) in raising cervical cancer awareness in the Vietnamese community [11]. Currently, there are no studies that evaluate the effectiveness of student pharmacists hosting seminars in promoting cervical cancer awareness, Pap smears and HPV vaccination among the Vietnamese communities. Positive results of this community educational intervention would call for the adoption of this educational model in pharmacy schools close to large Vietnamese-American populations.

## 2. Materials and methods

### 2.1. Population and protocol

Student pharmacists led cervical cancer awareness outreach efforts and hosted eight educational seminars at local community health fairs throughout San Diego in partnership with the Wesley First Methodist Church and the Vietnamese Federation. The health fairs were organized by student pharmacists of the University of California, San Diego Skaggs School of Pharmacy and Pharmaceutical Sciences. The health fairs were hosted in Vietnamese communities in San Diego and also offered free blood pressure, cholesterol and blood glucose measurements. A total of four health fairs were organized and two cervical awareness seminars were hosted at each health fair. The participants were given two screening questionnaires. The pre-questionnaire was given before the presentation; the same questionnaire was given after the intervention. The seminar consisted of a Microsoft PowerPoint presentation about the epidemiology, risk factors, signs and symptoms, diagnosis, and latest guidelines and prevention measure(s) related to cervical cancer (i.e., Pap smears and HPV vaccination). The seminars were presented in English with a Vietnamese interpreter. Everyone at the health fair was welcome to participate in the educational seminar. However, given the topic and targeted population, only Vietnamese women were asked to consent to participate in the study. To participate in the study, the participants had to be at least 18 years of age and an ethnically Vietnamese woman.

Both questionnaires were provided either in English or Vietnamese, depending on the participant's preference. Post-intervention questions required the participants to disclose information regarding demographics, such as the level of education, which was stated as high school level (9−12^th^) and beyond high school (community college and university). Other questions inquired about knowledge regarding cervical cancer, the reasons for not getting a Pap smear and the likelihood of obtaining Pap smear a year from the presentation. There were a total of eight questions that were the same in the pre- and post-questionnaires that compared the impact of the educational presentation on the participants.

### 2.2. Statistical analysis

Descriptive statistics were used to summarize the findings, a matched comparison (t-test) of the pre- and post-intervention questionnaires was used for statistical significance with a p-value <0.05.

### 2.3. Ethics approval of research

The study was approved by the UC San Diego Human Subject Research as per project summary number 121193. Further, the written informed consent was obtained from the respondents before participating in the seminar. The participants were able to leave the seminar at any time and the questionnaires were optional.

## 3. Results

A total of 120 Vietnamese women agreed to participate in the study. The demographics data showed that 97% of the participants were Vietnamese immigrants; 58% stated that they had trouble understanding English, 68% had a high school level education and 70% did not have health insurance (Table 1).

The percentage of Vietnamese women that previously had a Pap smear varied by age range.

Among the Vietnamese women, 77% of those aged 18−29 years reported having a Pap smear in the past as compared to 43% of the women aged 30−49 years and 46% of the women aged 50−69 years. There was an association between education level and Pap smears. Participants with a high school education were least likely to have received a Pap smear than participants with education beyond high school (43% vs. 67%, p = 0.004) (Table 1). Non-English speakers had a significant smaller percentage of Pap testing than English speakers (45% vs. 59%, p = 0.019). There was also a disparity noted among the participants without health insurance compared to those with health insurance (19% vs. 64%, p < 0.001). Finally, there seems to be no significant difference between Vietnamese immigrants and Vietnamese-American participants receiving a Pap smear (p = 0.390) (Table 1). The top five reasons for not obtaining a Pap smear included, unaware of where to go to obtain a Pap smear (24%), fear (16%), embarrassment (10%), might be painful or uncomfortable (9%) and a lack of a female doctor (5%); the no response rate was 17% (Table 2).

Participant knowledge about cervical cancer among Vietnamese women in general, causes of cervical cancer, signs and symptoms and prevention measures (i.e., Pap smears, HPV vaccination) significantly improved (p < 0.001) by post-intervention. Furthermore, there was a statistical significance in improvement among those who were planning to have a Pap smear in the next 12 months after the intervention (p = 0.002); 57% planned to have a Pap smear before the presentation versus 78% after the presentation.

After the intervention, the number of correct responses to the questions regarding age recommendations for Pap smears and whether Pap smears are the most effective at detecting cervical cancer confirmed statistically significant improvement (77% vs. 94%, p < 0.001 and 79% vs. 90%, p = 0.020, respectively).

**Table 1.** Baseline characteristics.

Population	Total	Participants who have had a Pap smear (%)		
	N = 120 (%)	Yes	No	Don't Know
**Age**				
18–29	22 (18%)	17 (77%)	5 (33%)	0 (0%)
30–49	28 (23%)	12 (43%)	12 (43%)	4 (14%)
50–69	70 (58%)	32 (46%)	22 (31%)	16 (23%)
**Nationality**				
Vietnamese-American	4 (3%)	3 (75%)	0 (0%)	1 (25%)
Vietnamese Immigrant	116 (97%)	58 (50%) P = 0.390	38 (33%)	20 (17%)
**Trouble Understanding English**				
Yes	69 (58%)	31 (45%)	21 (30%)	17 (25%)
No	51 (43%)	30 (59%) P = 0.019	18 (35%)	3 (6%)
**Education**				
High school (9^th^−12^th^)	81 (68%)	35 (43%)	27 (33%)	19 (81%)
Beyond high school (i.e., community college and university)	39 (32%)	26 (67%) P = 0.004	12 (31%)	1 (2%)
**Health Insurance**				
Yes	84 (70%)	54 (64%)	14 (17%)	16 (19%)
No	36 (30%)	7 (19%) P < 0.001	25 (69%)	4 (11%)

**Table 2.** Responses from participants that prevent them from having a Pap test.

Responses	N = 120
	Number	Percentage
Might be painful	19	9%
Fear	35	16%
Embarrassment	23	10%
Does not have a female doctor	10	5%
Does not believe in Pap smears	5	2%
Won't do any good	7	3%
Too costly	14	6%
Don't know where to go for a Pap smear	52	24%
Insurance won't pay for a Pap smear	1	<1%
Don't have time to see a doctor	9	4%
Had cervix removed (hysterectomy)	3	1%
Over 65 years old	5	2%
No response	38	17%

**Table 3.** Pre- and post-intervention among participants.

Questions		N = 120 (%)	N = 120 (%)
		Pre-Intervention	Post-Intervention
**Causes of cervical cancer**			
What causes cervical cancer?	Family or hereditary	24 (20%)	5 (4%)
	HPV	41 (34%)	112 (93%)
	Don't know	55 (46%)	3 (3%)
**Assess the knowledge on Pap smears**			
Do Vietnamese women have the highest risk of cervical cancer?	*Yes	73 (61%)	111 (93%)
	No	47 (39%)	9 (7%)
Do Vietnamese women have the lowest rate of Pap smears?	*Yes	65 (54%)	101 (84%)
	No	55 (46%)	19 (16%)
Do women with cervical cancer experience pain or discomfort?	Yes	80 (67%)	47 (39%)
	*No	40 (33%)	73 (61%)
Is it recommended that women over the age of 21 get a Pap smear?	*Yes	92 (77%)	113 (94%)
	No	28 (23%)	7 (6%)
Are Pap smears the most effective way of detecting cervical cancer?	*Yes	95 (79%)	108 (90%)
	No	25 (21%)	12 (10%)
Is the human papilloma virus (HPV) vaccination the most effective method of preventing cervical cancer?	*Yes	85 (71%)	114 (95%)
	No	35 (29%)	6 (5%)
Average percentage with P-value (*shows the correct answer)		62.8%	84.8% P = 0.001
Are you planning to have a Pap test in the next 12 months?	Yes	68 (57%)	93 (78%)
	No	52 (43%)	27 (22%)

## 4. Discussion

The finding of our current study reflects the findings of previous work from Gottvall et al. [7], who conducted HPV awareness outreach to the school-aged girls with the help of an online mode of education in a class setting. Further, another study was conducted by Kim et al. [8], showing that education regarding cervical cancer increases awareness in a way that is similar to our current study. The increased awareness of Pap smears according to age level could possibly be explained by differences in cultural attitudes between the USA and Vietnam. Sex education in San Diego schools begins in the 6^th^ grade and continues into the 12^th^ grade; this may explain the higher rate of Pap smears that occurs in the younger population, as there is more awareness in the USA [12]–[15]. Further, the language barrier may also contribute to fewer Pap smears, as well as a lack of health insurance [16]–[17]. Moreover, female doctors are difficult to contact due to the unavailability of facilities and living in a remote area. In fact, many women who participated in our seminar brought their family members to come and listen to our presentation. Therefore, one thing we could improve for future seminars is to include more information about where to obtain a Pap smear or the HPV vaccination.

A study was conducted by Nigerian health workers that identified the most important barriers to HPV vaccination, i.e., a lack of awareness (39%), vaccine availability (39%) and cost (13%) [18]. The current study also identified barriers that prevent participants from getting a Pap smear; the leading factors were no knowledge about Pap smears, fear, embarrassment, pain and the cost of testing. The Cervical Cancer Awareness project hosted by student pharmacists increased the awareness of cervical cancer and encouraged Vietnamese women to obtain a Pap smear within a year.

In our study, the number of women who planned to receive a Pap smear after the educational session significantly increased, but this was only a declarative statement; it would be interesting to follow these women to evaluate if they actually received a Pap smear following the educational program.

## 5. Limitations of study

The limitation of our project was that we were not able to obtain many participants for the amount of time and number of health fairs that we attended due to a lack of interest in participating. Most of them were more interested in getting their blood glucose, blood pressure and cholesterol levels checked rather than knowing about past HPV vaccination and cervical cancer information. As Vietnamese culture is very conservative, this topic is very sensitive and generally not to be discussed in the public. Therefore, many Vietnamese women do not want to participate in the educational presentation. In addition, the amount of time participants were willing to make allowances for the educational awareness was short.

## 6. Conclusion

The current study has highlighted the importance of outreach educational programs that help to increase the awareness about cervical cancer and prevention. Our study has shown the efficacy of student pharmacists' educational outreach in terms of educating the Vietnamese women about cervical cancer and improving their perceptions regarding HPV vaccination knowledge and Pap smear screening. Pharmacists, who are readily accessible in the community, could make a significant impact on public education about cervical cancer awareness and prevention, as well as have a positive impact on HPV vaccination rates. Overall, we hope this type of educational intervention seminar could be disseminated to underserved communities by pharmacy schools across the globe.

## References

[1] Wang SS, Carreon JD, Gomez SL (2010). Cervical cancer incidence among 6 asian ethnic groups in the United States, 1996 through 2004. Cancer.

[2] Singh GK, Jemal A (2017). Socioeconomic and Racial/Ethnic Disparities in Cancer Mortality, Incidence, and Survival in the United States, 1950-2014: Over Six Decades of Changing Patterns and Widening Inequalities. J Environ Public Health.

[3] US Cancer Statistics Working Group (2021). US Cancer Statistics Data Visualizations Tool, based on 2020 submission data (1999−2018): US Department of Health and Human Services, Centers for Disease Control and Prevention and National Cancer Institute.

[4] Stelzle D, Tanaka LF, Lee KK (2021). Estimates of the global burden of cervical cancer associated with HIV. Lancet Glob Health.

[5] Bosch FX, Manos MM, Muñoz N (1995). Prevalence of human papillomavirus in cervical cancer: a worldwide perspective. International biological study on cervical cancer (IBSCC) Study Group. J Natl Cancer Inst.

[6] Miller BA, Kolonel LN, Bernstein L (1996). Racial/Ethnic Patterns of Cancer in the United States 1988-1992.

[7] Gottvall M, Tydén T, Höglund AT (2010). Knowledge of human papillomavirus among high school students can be increased by an educational intervention. Int J STD AIDS.

[8] Kim HW, Lee YJ, Lee DB (2019). Effects of cervical cancer prevention education in middle-school girls in Korea: A mixed-method study. Heliyon.

[9] https://seer.cancer.gov/statfacts/html/cervix.html.

[10] Taylor VM, Nguyen TT (2008). Cervical cancer control research in Vietnamese American communities. Cancer Epidemiol Biomarkers Prev.

[11] Mock J, McPhee SJ, Nguyen T (2007). Effective lay health worker outreach and media-based education for promoting cervical cancer screening among Vietnamese American women. Am J Public Health.

[12] Bingham A, Drake JK, LaMontagne DS (2009). Sociocultural issues in the introduction of human papillomavirus vaccine in low-resource settings. Arch Pediatr Adolesc Med.

[13] Uzunlar Ö, Özyer Ş, Başer E (2013). A survey on human papillomavirus awareness and acceptance of vaccination among nursing students in a tertiary hospital in Ankara, Turkey. Vaccine.

[14] Donadiki EM, Jiménez-García R, Hernández-Barrera V (2013). Knowledge of the HPV vaccine and its association with vaccine uptake among female higher-education students in Greece. Hum Vaccin Immunother.

[15] Lee YS, Hofstetter CR, Irvin VL (2012). Korean American women's preventive health care practices: stratified samples in California, USA. Health Care Women Int.

[16] Ma GX, Gao W, Fang CY (2013). Health beliefs associated with cervical cancer screening among Vietnamese Americans. J Womens Health (Larchmt).

[17] De Alba I, Sweningson JM, Chandy C (2004). Impact of English language proficiency on receipt of pap smears among Hispanics. J Gen Intern Med.

[18] Nguyen NY, Okeke E, Anglemyer A (2020). Identifying Perceived Barriers to Human Papillomavirus Vaccination as a Preventative Strategy for Cervical Cancer in Nigeria. Ann Glob Health.

